# Explanatory models in neonatal intensive care: a tutorial

**DOI:** 10.1186/s41077-018-0085-2

**Published:** 2018-12-20

**Authors:** Willem W.L. van Meurs, Timothy A.J. Antonius

**Affiliations:** 1 Cardiovascular and Respiratory Physiology Group, Faculty of Science and Technology, University of Twente, Enschede, The Netherlands; 2 Department of Neonatology, Radboudumc Amalia Children’s Hospital, Nijmegen, The Netherlands; 3 Department of Pediatrics, Division of Neonatology, Radboud University Medical Center, P.O. Box 9101, 6500 HB Nijmegen, The Netherlands

## Abstract

**Background:**

Acute care providers intervening on fragile patients face many knowledge and information related challenges. Explanation based on causal chains of events has limitations when applied to complex physiological systems, and model-driven educational software may overwhelm the learner with information. We introduce a new concept and educational technology to facilitate understanding, reasoning, and communication in the clinical environment. The aim is to grasp complex physiology in a more intuitive way.

**Explanatory models (EM):**

An EM is a representation of relevant physiologic processes that provides insight into the relationships between therapeutic interventions and monitored variables, and their dependency on incidents and pathologies. We systematically analyze types of information incorporated into models and displayed in simulations and consider their explanatory relevance.

**Transposition of the great arteries (TGA):**

A conceptual model (diagram) of the normal neonatal cardiorespiratory system is adapted to reflect TGA and implemented in animated, interactive software.

**Illustration of educational use:**

The use of this model is illustrated via the explanation to pediatric residents of the relationships between blood pressures, blood flow rates, ventilation, oxygen saturation, and oxygen distribution in a neonate with TGA. Learners explore clinical scenarios and effects of therapeutic interventions.

**Discussion:**

Explanatory models hold promise as mental models for clinical practice and could possibly play a role in clinical decision making in neonatal intensive care and beyond.

**Companion software:**

The software is freely available via the web addendum: https://www.dropbox.com/sh/ciufq5rqxgs9bkt/AAC7oKsvkEr73eYUJkx0pZ1Ya?dl=0

## Introduction

Acute care providers intervene on fragile patients with incomplete information on underlying physiology. They frequently do so under time pressure, and diagnosis and choice of therapeutic interventions may have far reaching consequences for the patient. Resources include their knowledge of physiology and pharmacology, clinical experience, patient specific information from clinical signs, monitoring instruments and additional exams, and protocols. Communication with other members of the healthcare team and consultation of information sources expand this basis for intervention. Acute care physicians thus face major knowledge and information related challenges:

- Complexity of underlying (patho)physiology.
- Incomplete information on underlying (patho)physiology.
- Integration of general knowledge and patient specific information.
- Effective communication with co-workers about the patient.

We will argue in this tutorial that traditional explanation based on causal chains of events has limitations when applied to complex and partially unknown physiological systems. Model-driven simulations constitute an alternative form of explanation. Model-driven educational software [1–3] is often derived from models used for research and from this ancestry inherits a software implementation that presents the user with many options and much information, often in numerical format. This information may be meaningful in the research context but tends to overwhelm the learner in the educational context and is not conducive to understanding and practical use in an acute care context.

In an educational setting, we can take away the time pressure experienced in real or simulated clinical environments, but learning is still subject to the listed challenges. In this tutorial, we propose a technique and supporting technology aimed at pediatric residents and interns that can be used to facilitate understanding of complex conditions in neonatal intensive care. Our objective is to explain particular clinical situations, and to do so effectively and efficiently. This explanation is meant to facilitate reasoning and communication about similar cases in the clinical environment and to decide on appropriate therapeutic interventions. After a brief analysis of explanation, we present the concept of an explanatory model in acute care. We then present an explanatory model for transposition of the great arteries and a screen-based simulator implementing this model and illustrate how it is used in explanatory simulations. Evaluation of models and assessment of learning will be addressed in separate empirical studies.

## Explanatory models in acute care

In its basic form, an explanation is a valid deductive argument, based on true antecedent conditions and applicable general laws, that has as its conclusion that the phenomenon to be explained occurred [4]. Deductive explanation frequently takes the form of a causal chain of events. This form of explanation is not well suited for situations in acute care because chains of events do not adequately reflect complex underlying physiologic processes involving multiple variables, interactions, and feedback loops.

Alternatively, a phenomenon is explained by showing that it is generated by a model [5]. We define a model as a formal representation of the relationships between system quantities of interest and use simulation to show that the phenomenon in question occurs and how it is generated. We use the following working definition:

- An explanatory model in acute care is a representation of relevant physiologic processes that provides insight into the relationships between therapeutic interventions and monitored variables, and their dependency on incidents and pathologies.

To develop this concept further and to come to a working screen-based simulator, we analyze the different types of information that are incorporated into a model and displayed in simulations and consider their relevance in view of the explanatory purpose. As part of a model and simulator, design process [6] distinguishes an input-output description, and conceptual and mathematical models of the system under consideration.

The input-output description puts a boundary around the system under consideration. When applied to a physiologic system evolving in a clinical situation, it explicitly distinguishes the independent variables: disturbances and diagnostic and therapeutic interventions, from the dependent variables: clinical signs and monitored variables.

Conceptual models, often in the form of block or component diagrams, reflect qualitative, predominantly general information about the system under consideration. A block diagram reflects subsystems, key variables, and causal relationships between subsystems. A component diagram reflects network structure and interactions between components. In the discussion, we will come back to the limitations of this mechanistic, engineering approach to modeling of naturally occurring systems.

**Fig. 1:**
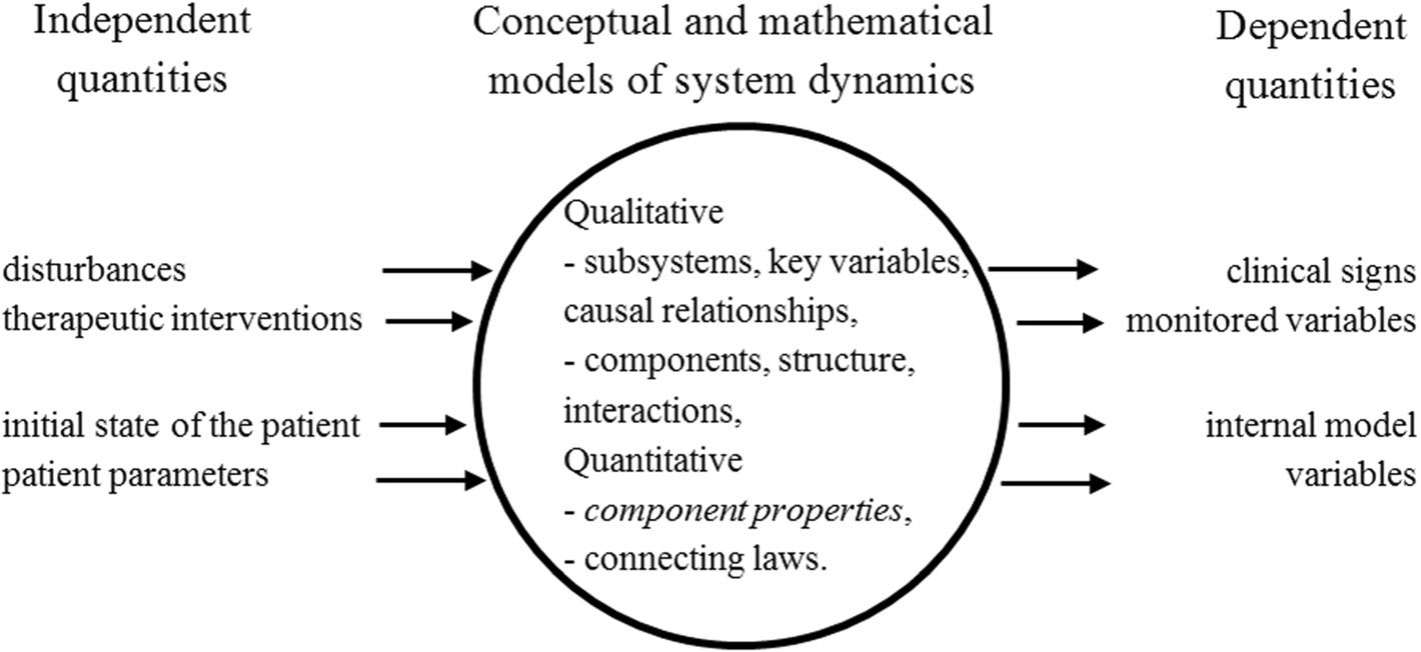
Types of information incorporated into an explanatory model. This includes variables and parameters that can be manipulated and displayed in simulations. Patient-specific information is included in component properties; other information is mostly general

This qualitative information gets incorporated into a mathematical model, adding quantitative information in the form of component properties and connecting laws. The former often takes the form of patient specific constants, called patient parameters. In a mathematical model, the quantities representing the physiologic state of the patient are often made explicit. For a given clinical situation, the initial state is part of the independent quantities. Selected patient parameters can be manipulated and selected internal model variables can be displayed. Figure 1 summarizes the different types of information that can be incorporated into an explanatory model.

We carefully select the independent and dependent quantities that are relevant in the particular discussed situation to avoid overwhelming the learner with material that is irrelevant for explanation. By making the simulation interactive, extrapolation to clinical decision making and actual therapeutic intervention is facilitated. By providing access to selected system parameters we further expand the experiential, interventionist dimension of explanation; manipulation of those parameters and observing the dynamic effects in real-time result in a powerful learning experience.

Due to their visual nature, conceptual models have a high explanatory impact and can be made to represent a level of detail that is appropriate for the system under consideration and yet simple enough to be of use in acute situations, including for dialog with colleagues. Downsides to conceptual models, when considered as explanatory models, include that they are static, even though meant to represent dynamic systems and processes, are limited to qualitative aspects of system operation, and are frequently equivocal and incomplete. We address these shortcomings by using animations of model structure, and of component sizes and colors in proportion to the values of critical quantities, adding dynamic graphs of selected variables, and by maintaining a careful mapping between underlying mathematical and animated conceptual models.

For our target audience, the explanatory relevance of the remaining aspects of mathematical models is limited. We therefore shield many internal model quantities and all equations and their software implementation from the learner.

## Transposition of the great arteries

**Fig. 2:**
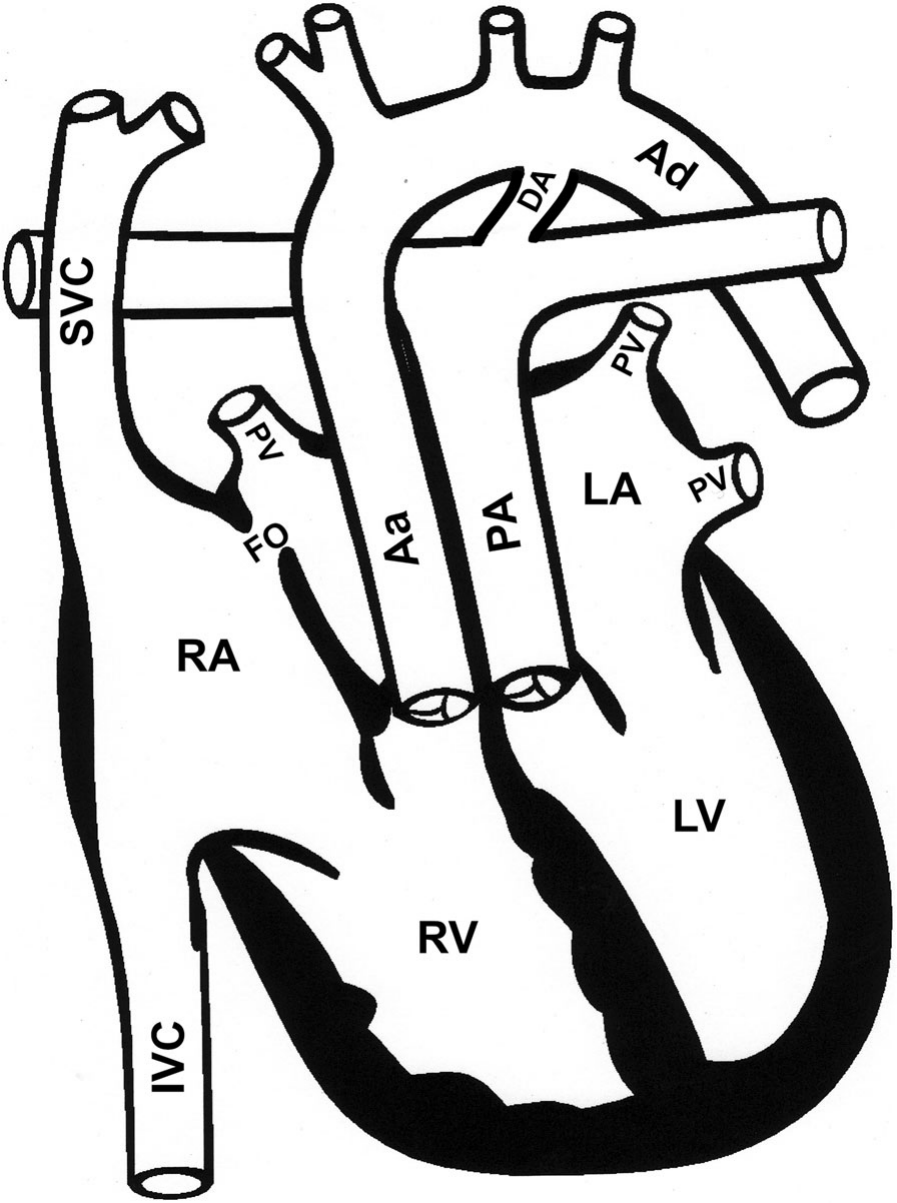
Schematic representation of a transposition of the great arteries. Pulmonary veins (PV), left atrium (LA), left ventricle (LV), ascending aorta (Aa), descending aorta (Ad), inferior vena cava (IVC), superior vena cava (SVC), right atrium (RA), right ventricle (RV), pulmonary arteries (PA), ductus arteriosus (DA), foramen ovale (FO)

The interactive class on complex clinical situations in neonatal intensive care is aimed at pediatric residents and interns. In this tutorial, we focus on explaining the underlying physiology and management options in a single complex condition, namely transposition of the great arteries (TGA) with ventricular septal defect (VSD), one of the more common complex congenital heart lesions in the newborn [7, 8]. In this defect, the vessels that carry blood to the lungs and to the body are improperly connected (Fig. 2). The pulmonary artery is connected to the left ventricle and the ascending aorta to the right ventricle. Oxygenated blood from the left ventricle returns to the lungs, and deoxygenated blood is carried to the tissues. Two connections (shunts) are required to make sure these two separate circulations can mix so oxygenated blood can be supplied to the body. The ductus arteriosus is a shunt which normally closes after birth. Affected babies are admitted to the ICU and given medication (prostaglandin E1) to make sure the ductus stays patent. Sometimes, a procedure called a balloon atrial septostomy is necessary to enlarge another shunt, the foramen ovale, to further improve mixing of oxygenated and deoxygenated blood. Surgical correction within 1–2 weeks after birth is often necessary to restore normal anatomy. Morbidity and mortality in this condition therefore depend on oxygen flows over these two shunts. Oxygen flow depends on blood flow and on oxygen content. Blood flow depends on systemic and pulmonary pressures and resistances, yet also affects the driving pressures. Partial pressures driving oxygen transport are in the same time affected by that transport, and by metabolism. A number of active control systems further complicate simple causal reasoning about the involved phenomena, making them a challenging to understand using traditional means.

**Fig. 3:**
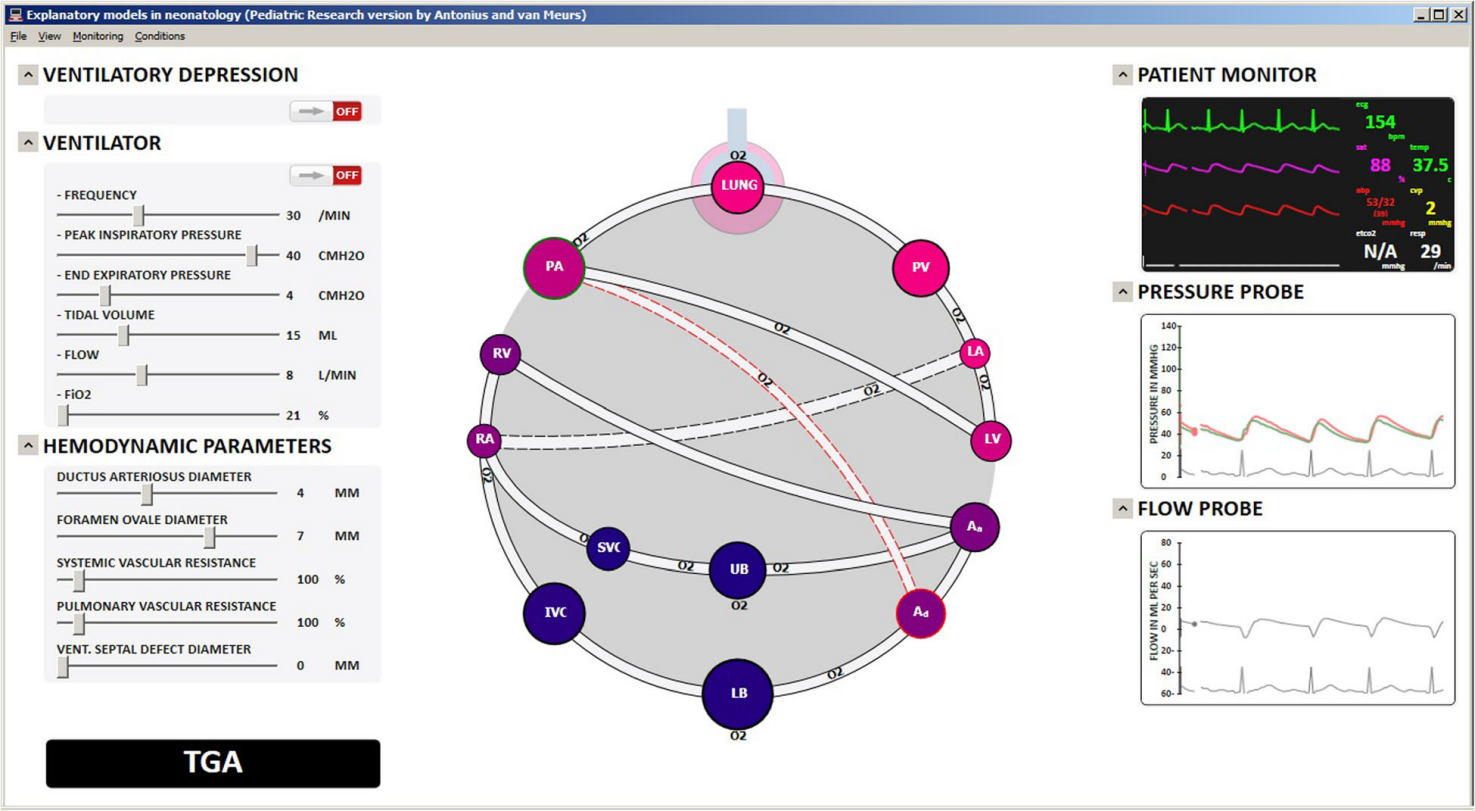
Explanatory model for transposition of the great arteries (screen shot). Pulmonary veins (PV), left atrium (LA), left ventricle (LV), ascending aorta (Aa), descending aorta (Ad), lower body (LB), upper body (UB), inferior vena cava (IVC), superior vena cava (SVC), right atrium (RA), right ventricle (RV), pulmonary arteries (PA)

Software implementing the explanatory model described below is freely available via the web addendum to this tutorial: https://www.dropbox.com/sh/ciufq5rqxgs9bkt/AAC7oKsvkEr73eYUJkx0pZ1Ya?dl=0. A screen shot of the main trainee interface is presented in Fig. 3.

The represented situation is TGA with a patent ductus arteriosus (DA) and open foramen ovale (FO). VSD is initialized to zero. The animated conceptual model of the cardiorespiratory system is presented in the center of the screen. It results from adaptations of a hydraulic analog used by Goodwin et al. [9] and its expansions to include the shunts of the neonatal circulation and VSD [10, 11]. New elements introduced to make it fit the explanatory objectives include:

1. Simplified components, including removal of valves and resistances.
2. Ascending and descending aorta, upper and lower body, and superior and inferior vena cava, c.f. Huikeshoven [12].
3. Variable diameter of shunts reflected in the width of the respective lines. In keeping with Poiseuille’s law, modeled resistance is proportional to the fourth power of the diameter.
4. Variable systemic vascular resistance (SVR) and pulmonary vascular resistance (PVR) again reflected in the width of the lines.
5. Animated volume dependent radius of the lumped alveolar space, allowing for animation of ventilation. Space diameter is proportional to the cube root of alveolar volume.
6. Animated volume dependent radii of lumped vessels providing information on blood volume distribution and allowing for animation of pulsatile behavior. Vessel diameter is proportional to the cube root of blood volume.
7. Animated oxygen saturation via traditional color coding of the vessels. The conceptual model reflects a one-to-one match between lumped blood vessels and gas compartments, c.f. Beneken and Rideout [13].
8. Animated oxygen uptake by the pulmonary blood, distribution via blood vessels, and metabolism in upper and lower body. Velocity of O2 symbol movement is proportional to instantaneous oxygen transport. When present, pulsatile and reverse flows are reflected in the movement.

Animations can only be observed by running the software. Several causal relationships are implicitly reflected in these animations, notably the dependency of oxygen distribution on ventilation and contractile behavior of the heart chambers, and on oxygen saturation in the upstream compartment. In steady state, oxygen flow rates into and out of a particular compartment, including lung uptake and tissue metabolism, match. Influences of baro- and chemo reflexes on respiratory and heart rates are included, but not visually emphasized for explanation of this particular condition.

To the upper-right of the animated conceptual model a clinical monitor emulator is displayed. Pressure and flow probes displayed to the bottom-right of the animated model provide the possibility to investigate variables that are not routinely monitored, but that are critical for explaining the complex interdependencies of blood volumes, pressures, and flow rates. By clicking on any segment of the circulation, upstream and downstream pressure and flow rate through the segment are displayed. This is particularly useful to show the pressures across and flow rates through the three shunts. In Fig. 3, the tracings correspond to the ductus arteriosus, which is highlighted in the conceptual model.

One disturbance and several potential therapeutic interventions are displayed to the upper-left of the animated conceptual model. Figure 3 shows the ventilatory depression and the ventilator emulator used in explanation of TGA. Selected patient parameters can be modified via controls at the bottom-left of the animated conceptual model. Interventions are also displayed in the animated conceptual model, and consequences are computed and displayed in real-time.

The mathematical model consists of coupled cardiovascular, respiratory gas transport, autonomic nervous system (baro- and chemoreceptors), lung- and chest wall mechanics, pulmonary gas exchange, and myocardial oxygen balance models [14]. The cardiovascular and baroreflex models are based on Goodwin [9] and adapted and expanded to reflect the neonatal circulation [10, 12, 15]. The oxygen transport model is based on van Meurs [6] and Sá Couto [16]. Models and interfaces were implemented in C# by the second author. Although a full model validation is not part of this tutorial on the concept of explanatory models, we compared the selected critical model outputs: oxygen saturations and blood pressures to those reported in literature [17, 18]. In the scenario where there is a large PDA (3.5 mm) and a normal, non-restrictive FO (5.5 mm diameter), the oxygen saturations are in the range of the reported normal values for TGA without VSD (75–85%). Simulated oxygen saturations are found to be highly dependent on FO and PDA sizes, which correspond to clinical experience. The shunt direction across the PDA is dependent on the pulmonary and systemic vascular resistances; with normal resistances the shunt direction is mainly from the aorta to the pulmonary artery, which again corresponds to data reported in the literature [17]. In the presence of a VSD (3 mm), there is improved mixing, resulting in higher simulated oxygen saturations (> 80%), again as expected and described in literature [17, 18].

## Illustration of educational use

There are several ways in which the described explanatory model can be used. Here, we describe a 2-h class for 6–10 pediatric residents. The goal is to explain the complex relationships between ventilation, blood pressures and flow rates, and oxygen distribution in a neonate with TGA. The faculty has two laptop computers at his or her disposition, both running the software.

The class starts with the software running a model of the normal neonatal circulation with FO and DA shunts. The different compartments are described by the faculty. In our experience, the students adapt very quickly to this representation of the circulation and oxygen distribution.

The ventilator emulator, Fig. 3, is introduced. As much as possible, interventions in underlying physiology are introduced via real therapeutic interventions, e.g., balloon septostomy widening the FO, or norepinephrine injection increasing SVR. A case of high pulmonary vascular pressure is used to highlight the location of clinically monitored and underlying physiologic variables on the screen and to demonstrate the simulated dynamic consequences of interventions.

After this “refresher” on the normal neonatal circulation, also introducing the software, a static anatomical diagram of a heart with TGA is presented and discussed by the faculty. Then, one of the key concepts in TGA, namely the importance of FO and DA for adequate perfusion and oxygenation, is discussed and demonstrated in the explanatory model by the faculty.

Two groups of 3–5 students are then invited to use the software to examine effects of specific interventions and composite clinical situations on blood pressures, flow rates and oxygen delivery. Both groups are asked to explore the effects of FO and DA size. Then each group is asked to explore one of two scenarios: pharmacological interventions affecting SVR and PVR, and initiation of artificial ventilation increasing intrathoracic pressure. After 30 min, the faculty invites each group to explain their findings to the other group, using the model.

In conclusion, the presence or absence of ventricular septal defect (VSD) and its effects are presented by the faculty, using the explanatory model, and building upon the earlier obtained insights.

## Discussion and conclusions

Mechanistic, engineering approaches to modeling of naturally occurring systems have their limitations; our bodies are not made out of neatly separated interacting systems and subsystems, and discrete components. However, the analogies and traceable simplifications inherent to this approach facilitate the use of resulting models as explanatory models.

The explanatory model introduced in this tutorial combines a systems perspective, animated conceptual models, and an interactive simulation approach. This sets it apart from research-oriented modeling studies, focusing primarily on accurate descriptive or predictive mathematical models of a particular aspect of human physiology. The systems perspective and animated conceptual models capture relevant general knowledge and patient specific information. The interactive, model-driven simulation approach uses this knowledge and information to explore the effects of therapeutic interventions and differences among patients and conditions, in order to explain clinically observable outcomes. We illustrate the use of this model in the explanation to pediatric residents of the complex relationships between pressures, flow rates, and oxygen distribution in a neonate with TGA.

General knowledge of the human cardiorespiratory system is predominantly contained in the conceptual model and in connecting laws. Information about the specific patient is predominantly located in the numerical values of parameters and initial states in the mathematical model. The cardiorespiratory systems of humans of all ages, and indeed of different mammal species, can be characterized simply by changing these values, but leaving the conceptual model intact. Many pathologies are also reflected by changing parameter values. The fetal and early neonatal cardiorespiratory systems are notable exceptions: their structure, part of the conceptual model, differs considerably from the mature system, and changes dramatically during and shortly after birth. Pathologies also affect structure, rather than only parameter values. Understanding the physiology of these “different animals” therefore represents particular challenges, which we attempt to address with the presented explanatory model.

The current model is able to reflect most of the common congenital heart diseases and can be expanded to include respiratory conditions. Note that this results in an important economy of effort, for model and software developers, for learners, and ultimately for clinical practitioners. The same approach can be used to explain complex situations in intensive care of other pediatric and adult patients. The possibility to adapt the physiology to reflect specific patients facilitates its use in clinical case discussions. Due to their visual nature and to the carefully designed balance between model simplicity and descriptive and predictive powers, explanatory models hold promise as mental models for clinical practice and could possibly play a role in clinical decision making. These real-world applications greatly depend on the ease with which stored knowledge can be handled within (visual) working memory [19]. Their use to reflect the effects of clinical interventions “on the fly” and help predict outcomes could also be explored.

In this tutorial, we define the concept of an explanatory model in acute care, present an explanatory model for transposition of the great arteries, illustrate its educational use, and discuss further uses of the concept.

## Abbreviations

DA: Ductus arteriosus
EM: Explanatory models
FO: Foramen ovale
PVR: Pulmonary vascular resistance
SVR: Systemic vascular resistance
TGA: Transposition of the great arteries
VSD: Ventricular septal defect
